# PKM2 Inhibitors Induce Autophagic Cell Death Through Suppression of PKM2-Mediated Glycolysis in Cisplatin-Resistant Ovarian Cancer Cells

**DOI:** 10.3390/ijms27157058

**Published:** 2026-08-06

**Authors:** Hae Eun Park, Haeun Lee, Ju Ri Kim, Eunah Lee, Jae Hyeon Park, Hyung Sik Kim

**Affiliations:** School of Pharmacy, Sungkyunkwan University (SKKU), 2066, Seobu-ro, Suwon 16419, Gyunggi-do, Republic of Korea

**Keywords:** shikonin, pyruvate kinase M2, cisplatin resistance, ovarian cancer, glycolysis pathway

## Abstract

Ovarian cancer is among the most lethal gynecological malignancies due to its poor prognosis and lack of early symptoms. Cisplatin remains the primary chemotherapeutic agent; however, resistance to cisplatin in advanced ovarian cancer is a major cause of treatment failure. Pyruvate kinase M2 (PKM2) is markedly upregulated in ovarian cancer tissues and contributes to cisplatin resistance, though its therapeutic relevance has not been fully defined. This study investigated whether shikonin and compound 3K, both PKM2 inhibitors, could enhance anticancer effects in cisplatin-resistant SKOV-3 cells by modulating autophagic pathways. Cytotoxicity assays revealed that treatment with shikonin or compound 3K significantly reduced PKM2 expression. Combination therapy with high-dose PKM2 inhibitors and cisplatin increased apoptosis compared to controls, although the modest induction suggests apoptosis is only partially responsible for the observed effects. Additionally, Shikonin and compound 3K treatment suppressed PKM2-mediated glycolysis and induced autophagic cell death in cisplatin-resistant ovarian cancer cells, as evidenced by increased LC3-II expression, autophagosome formation, and reduced cell viability. These findings indicate that PKM2 overexpression plays a central role in cisplatin resistance in ovarian cancer. Targeting PKM2 with inhibitors such as shikonin or compound 3K may represent a promising strategy to overcome chemoresistance and improve therapeutic outcomes in patients with advanced ovarian cancer. These findings strongly suggest that PKM2 overexpression plays a key role in cisplatin resistance in ovarian cancer. Thus, PKM2 inhibitors use may be a highly effective strategy for overcoming chemoresistance and improving outcomes in patients with advanced ovarian cancer.

## 1. Introduction

Ovarian cancer is often referred to as a silent killer because of the difficulties in early diagnosis and treatment. According to the latest estimates from the American Cancer Society, approximately 2,114,850 new cancer cases and 626,140 cancer-related deaths are expected to occur in the United States in 2026, underscoring the persistent global burden of cancer despite significant advances in diagnosis and treatment [1]. The symptoms of ovarian cancer, including abdominal bloating, indigestion, and lower abdominal pain, are often mistaken for non-cancer-related issues [2]. Consequently, ovarian cancer is typically diagnosed at advanced stages (stage 3 or 4). Studies have shown that the survival rate is significantly lower when ovarian cancer is diagnosed at advanced stages than at earlier stages 1 and 2 [3].

Although ovarian cancer is not as prevalent as other cancers, its high mortality rate underscores the importance of research focusing on effective diagnosis and treatment. In addition to cytoreductive surgery and platinum-based chemotherapy, poly (ADP-ribose) polymerase (PARP) inhibitors have emerged as an important therapeutic strategy for ovarian cancer [4]. In particular, patients with BRCA mutations or homologous recombination deficiency derive substantial clinical benefit from PARP inhibitor therapy, with significant improvements in progression-free survival and outcomes during maintenance treatment [4,5]. Currently, a combination of pharmacological therapy with paclitaxel and carboplatin is the standard treatment approach [6]. However, a significant challenge in treatment is the development of drug resistance, particularly when ovarian cancer progresses and patients continue to be exposed to platinum-based drugs [7,8]. To increase anticancer efficacy on ovarian cancer, long-term administration at high concentrations is one potential strategy [9,10]. However, this approach often leads to side effects, including kidney toxicity and gastrointestinal issues. Therefore, drug delivery strategies has focused on enhancing the anticancer effects of these chemotherapeutic drugs without increasing their concentrations [11]. Cisplatin is typically used as the primary drug for treating patients with ovarian cancer. It interacts with nucleophilic components, such as ribosomal RNA, spliceosomes, and telomerase, accumulating not only in the nucleus but also in the mitochondria, cell membrane, and endoplasmic reticulum [12,13]. This accumulation disrupts the double-helical DNA structure, causing DNA damage, inhibiting DNA replication, and inducing G2 phase cell cycle arrest, ultimately leading to apoptosis [14].

Pyruvate kinase M2 (PKM2), a key regulator of cellular metabolism, is involved in the final step of glycolysis where it converts phosphoenolpyruvate into pyruvate [15,16]. PKM2 exists in two forms: a dimer and a tetramer, with the tetramer being the active form. In its dimeric form, PKM2 regulates the rate-limiting step of glycolysis, shifting glucose metabolism from the normal oxidative phosphorylation pathway to lactate production, which is a hallmark of tumor metabolism [16]. Therefore, a potent and selective PKM2 inhibitor was designed to directly target overexpressed PKM2. Previous studies have demonstrated that modulation of the AKT/AMPK/mTOR signaling pathway plays a critical role in the regulation of autophagy and cellular metabolism in cancer cells, thereby contributing to the antitumor effects of metabolic-targeting agents [17,18].

Compound 3K is a potent small-molecule PKM2 inhibitor developed through structure-based optimization of dithiocarbamate-substituted naphthoquinone derivatives [19]. Previous our study has demonstrated that compound 3K markedly inhibits cell proliferation and colony formation while significantly suppressing xenograft tumor growth in ovarian cancer models without inducing apparent systemic toxicity [20,21]. These findings indicate that targeting PKM2 with compound 3K effectively disrupts cancer metabolic reprogramming, supporting its potential as a novel therapeutic strategy for PKM2-dependent cancers. Shikonin, a natural product derived from the roots of the perennial herb *Lithospermum erythrorhizon*, possesses wound-healing, antibacterial, anti-inflammatory, and antitumor properties [22,23]. Extensive research on shikonin has explored its potential as an anticancer agent, with its mechanism of action linked to multiple pathways involved in targeting the PI3K/AKT signaling pathway [24,25].

SKOV-3 cells are a cisplatin-resistant ovarian cancer cell line characterized by the absence of functional p53 expression (p53-null phenotype), making them a useful model for investigating therapeutic strategies against treatment-resistant ovarian cancer [26,27]. Several approaches have been explored to overcome cisplatin resistance in ovarian cancer, including combination therapies with targeted agents, regulation of apoptosis and autophagy pathways, and inhibition of metabolic reprogramming such as PKM2-mediated glycolysis [28,29,30]. These strategies aim to restore chemosensitivity and improve treatment outcomes. Given the enhanced anticancer effects observed following combined treatment with cisplatin and PKM2 inhibitors, we next investigated whether these effects were associated with changes in cellular migratory capacity.

## 2. Results

### 2.1. Targeting PKM2 Inhibition Effectively Reduced SKOV-3 Cell Proliferation

The chemical structures of compound 3k and shikonin are shown in Figure 1A,B. In this study, compound 3K was used as a positive PKM2 inhibitor. Upon entry into the cells, cisplatin is hydrated and becomes positively charged (Figure 1C). This activated form binds to purine bases in DNA, forming DNA adducts that interfere with DNA replication and transcription, ultimately leading to cell death.

To evaluate the effectiveness of targeting PKM2 in SKOV-3 cells (Figure 1D), PKM2 expression levels were assessed in various ovarian cancer cell lines. In addition to SKOV-3, PKM2 expression levels were analyzed in other ovarian cancer cell lines, including TOV-21G, CAOV-3, and OVCAR-3, using Western blotting (Figure 2A). Data presents a bar graph quantifying the band intensities. The results indicated that PKM2 expression was the highest in SKOV-3 cells, followed by those in TOV-21G, CAOV-3, and OVCAR-3 cells, respectively. PKM2 expression was elevated in SKOV-3 cells, suggesting a potential association between PKM2 expression and the biological characteristics of these cells. However, protein expression alone does not establish a causal role in cell growth (Figure 2B). Targeting PKM2 in SKOV-3 cells may enhance sensitivity to anticancer drugs.

### 2.2. Cytotoxicity of PKM2 Inhibitors in Cisplatin-Resistant SKOV-3 Cells

To characterize the cisplatin-resistant ovarian cancer cell line SKOV-3, we examined PKM2 expression and found that it was markedly elevated, suggesting a potential association between PKM2 expression and the biological characteristics of cisplatin-resistant cells. However, elevated PKM2 expression alone does not establish a causal role in cell growth or cisplatin resistance. To characterize the drug sensitivity of the ovarian cancer cell lines, we determined their responses to cisplatin. The IC_50_ values for SKOV-3 and OVCAR-3 were 56.84 and 15.97 μM, respectively, indicating that SKOV-3 is substantially more resistant to cisplatin than OVCAR-3 (Figure 3A).

To determine whether PKM2 inhibition enhances cisplatin sensitivity, cell viability was compared between cisplatin-treated cells and cells co-treated with cisplatin and PKM2 inhibitors at each corresponding cisplatin concentration. As shown in Figure 3B, treatment with compound 3K alone reduced cell viability by approximately 10% at 10 μM, but this effect was not statistically significant. In contrast, co-treatment with cisplatin significantly enhanced cytotoxicity, with a significant reduction in cell viability observed at Compound 3K concentrations of 4 μM and above. As shown in Figure 3C, shikonin alone inhibited SKOV-3 cell viability in a dose-dependent manner, with significant cytotoxicity observed at concentrations of 2 μM or higher. However, when combined with cisplatin, a significant reduction in cell viability was observed at a lower shikonin concentration (1 μM), and the combination produced substantially greater cytotoxicity than shikonin alone across the tested concentration range. These results suggest that shikonin enhances the sensitivity of SKOV-3 cells to cisplatin. Similar to Compound 3K, co-treatment with shikonin and cisplatin exhibited greater anticancer activity in SKOV-3 cells than either treatment alone. Both PKM2 inhibitors significantly enhanced the cytotoxic effects of cisplatin, suggesting that PKM2 inhibition may sensitize SKOV-3 cells to cisplatin and could represent a potential therapeutic strategy for overcoming cisplatin resistance in ovarian cancer.

### 2.3. Combination Treatment with PKM2 Inhibitors and Cisplatin Induces Cell Cycle G2/M Phase Arrest

Cell cycle analysis was conducted to examine drug treatment-induced cell cycle arrest. Histograms analyzed by flow cytometry showed a G2/M phase arrest (Figure 4A,B). The bar graphs display the proportion of cells in the G1, S, and G2/M phases, with the combination treatment groups indicated by the slashed bars (Figure 4C,D). Both Compound 3K and shikonin induced G2/M phase arrest in single-treatment and combination-treatment groups. While co-treatment with cisplatin resulted in a greater proportion of cells in the G2/M phase than either agent alone, but the increase was not significant. Therefore, these findings should be interpreted with caution and are not intended to suggest a strong synergistic effect on cell cycle arrest (Figure 4C,D). This suggests that PKM2 inhibition enhances the anticancer activity of cisplatin by promoting a higher level of cell cycle arrest.

### 2.4. Cisplatin Treatment with PKM2 Inhibitors Induces Apoptosis in SKOV-3 Cells

The rate of apoptotic cell death following the combination treatment was assessed using Annexin V–FITC/PI staining and flow cytometry. After the drug treatment, significant morphological changes were observed in the cells. Compared with the single-treatment groups, treatment with PKM2 inhibitors resulted in a marked reduction in cell density and confluency, accompanied by an increase in detached and rounded cells, indicating decreased cell viability (Figure 5A). Apoptosis was further analyzed using flow cytometry following treatment with PKM2 inhibitors and cisplatin (Figure 5B,C). The highest-dose combination of PKM2 inhibitors and cisplatin resulted in a statistically significant increase in the apoptotic cell population compared with the control group. However, the magnitude of apoptosis induction was relatively modest, suggesting that apoptosis contributes only partially to the anticancer effects of the PKM2 inhibitor–cisplatin combination. Therefore, additional cell death mechanisms are likely involved in mediating the overall therapeutic response. The bar graphs in Figure 5D,E summarize the percentages of early and late apoptotic cell death. Compared with the rate observed in single treatments, the combination of PKM2 inhibitors and cisplatin significantly increased the rate of apoptotic cell death.

### 2.5. PKM2 Inhibitors Regulate the Autophagy Pathway in SKOV-3 Cells

An acridine orange (AO) fluorescence staining assay was performed to evaluate autophagy induced by Compound 3K and shikonin. Acidic vesicular organelles (AVOs), including autophagosomes and autolysosomes, were detected by the accumulation of orange fluorescence, indicating the induction of autophagic activity. Autophagosome formation in SKOV-3 cells was evaluated following treatment with Compound 3K or shikonin in combination with cisplatin (Figure 6A,B). The combination treatment groups exhibited a greater accumulation of acidic vesicular organelles than the corresponding single-treatment groups. Both shikonin and Compound 3K induced autophagic responses in SKOV-3 cells, and the combination with cisplatin further enhanced autophagosome formation.

### 2.6. Effect of Cisplatin and PKM2 Inhibitors on SKOV-3 Cell Metastasis

To assess the impact of PKM2 inhibitors and cisplatin on SKOV-3 cell migration, a cell migration assay was conducted. Cells were seeded in a 96-well plate, scratched to create a wound, and cell migration was monitored under a microscope. The results showed that the cell migration rate was significantly lower in the combination treatment of compound 3K and shikonin than in the single-drug treatment group (Figure 7A,B). The graph illustrates the wound closure area as a percentage of the total scratch area, with 100% indicating complete wound closure. When observed at 12 h intervals over 24 h, the control group exhibited complete closure (100%), while the highest concentration group showed approximately 50% closure. This indicated a significant reduction in cell migration ability in the combination treatment group, demonstrating the enhanced effect of PKM2 inhibitors in combination with cisplatin (Figure 7C,D).

### 2.7. PKM2 Inhibition Influences Glycolytic Functional Stress, as Assessed by ECAR Measurements

Extracellular acidification was measured using the Seahorse assay to evaluate glycolytic functional stress. The glycolysis pathway was assessed by sequentially adding glucose, oligomycin, and 2-DG to the cells and altering the conditions to identify the changes in glycolysis (Figure 8A,C) Combination treatment with cisplatin and shikonin, or cisplatin and Compound 3K, resulted in significantly greater anticancer effects than treatment with either agent alone. Glycolytic stress was present in the single-drug treatment group but was significantly increased in the combination treatment group. The data from Figure 8B are represented as bar graphs, categorizing non-glycolytic acidification, glycolysis, glycolytic capacity, and glycolytic reserves. Both glycolysis and glycolytic capacity decreased in the combination treatment groups. Similar to compound 3 K, the combination treatment with shikonin and cisplatin induced greater glycolytic functional stress than single-drug treatment (Figure 8D).

## 3. Discussion

This study demonstrates the potential of targeting PKM2 as a therapeutic strategy for regulating cell proliferation and overcoming cisplatin resistance in SKOV-3 ovarian cancer cells. SKOV-3 cells are a cisplatin-resistant ovarian cancer cell line characterized by the absence of functional p53 expression (p53-null phenotype), making them a useful model for investigating therapeutic strategies against treatment-resistant ovarian cancer [28,31,32]. However, this protective mechanism is impaired in SKOV-3 cells with p53-null type, indicating that even cell damage by cisplatin does not significantly affect their survival, proliferation, or metastasis. This contributes to the drug resistance observed in SKOV-3 cells.

PKM2, a key enzyme in the glycolytic pathway, is implicated in cancer cell metabolism and proliferation [33]. Our data showed that PKM2 expression was significantly higher in SKOV-3 cells than in the other ovarian cancer cell lines examined (TOV-21G, CAOV-3, and OVCAR-3), suggesting a potential association between elevated PKM2 expression and the cisplatin-resistant phenotype of SKOV-3 cells. Previous studies have demonstrated that PKM2 contributes to cisplatin resistance in ovarian cancer, gemcitabine resistance in pancreatic cancer, and oxaliplatin resistance in colorectal cancer [34,35]. Our findings indicate that PKM2 inhibition suppresses the growth of cisplatin-resistant SKOV-3 cells and enhances their sensitivity to cisplatin. Although this study was limited to the SKOV-3 cell line, our findings support the potential of PKM2 as a therapeutic target in cisplatin-resistant ovarian cancer. Future studies using additional ovarian cancer cell lines with diverse molecular characteristics, as well as in vivo models, are needed to determine the broader applicability of these findings. Overall, these results provide a rationale for further investigation of PKM2-targeted strategies to overcome cisplatin resistance in ovarian cancer.

Compound 3K and shikonin modestly altered cell-cycle distribution, particularly by increasing the proportion of cells in the G2/M phase; however, these changes were insufficient to establish definitive G2/M cell-cycle arrest. Nevertheless, previous studies have suggested that disruption of G2/M progression may enhance the cellular response to DNA damage induced by chemotherapeutic agents [36,37]. The modest increase in the G2/M cell population may be associated with PKM2 inhibition-induced oxidative stress and mitochondrial dysfunction. Previous studies have demonstrated that PKM2 plays a critical role in regulating mitochondrial metabolism and cellular redox homeostasis, providing a potential mechanistic basis for these observations [38,39]. Combination treatment with PKM2 inhibitors and cisplatin significantly reduced the migratory capacity of SKOV-3 cells. Migration assays showed that cells treated with the combination exhibited significantly lower migratory ability than those treated with either treatment alone.

This study also investigated the potential role of PKM2 in cisplatin-resistant ovarian cancer using the SKOV-3 cells, as cisplatin resistance is a common challenge in advanced ovarian cancer. Our results demonstrated that SKOV-3 cells exhibited a substantially higher IC_50_ value for cisplatin than OVCAR-3 cells, indicating a greater degree of cisplatin resistance (Figure 3A). In the present study, co-treatment with shikonin and cisplatin produced a greater reduction in cell viability than either treatment alone, suggesting that PKM2 inhibition enhances the anticancer activity of cisplatin and may contribute to overcoming cisplatin resistance. Further investigation of the underlying mechanisms revealed that combination treatment induced a significantly greater level of apoptosis than either treatment alone, as demonstrated by flow cytometric analysis with Annexin V-FITC/PI staining. Shikonin alone did not significantly induce apoptosis, whereas when combined with cisplatin, it significantly increased apoptotic cell populations in a dose-dependent manner. These findings suggest that PKM2 inhibition enhances the susceptibility of SKOV-3 cells to cisplatin-induced apoptosis. These results suggest that PKM2 contributes to cellular survival mechanisms and that its inhibition enhances the pro-apoptotic effects of cisplatin in SKOV-3 cells.

We investigated the effects of PKM2 inhibition on autophagy in SKOV-3 cells. Autophagy, a cellular process that maintains homeostasis, plays a dual role in cancer by promoting either cell survival or cell death under stressful conditions [40,41]. Both shikonin and compound 3K induced autophagic responses in SKOV-3 cells; however, the molecular mechanisms underlying these effects were not investigated in the present study and warrant further investigation. Our results showed that, when combined with cisplatin, shikonin significantly increased autophagosome formation. These findings suggest that PKM2 inhibition enhances autophagic responses during cisplatin treatment, although whether this response contributes to autophagic cell death remains to be determined.

The migration capacity observed following combination treatment suggests that PKM2 inhibition enhances the sensitivity of SKOV-3 cells to cisplatin. Because cell migration is associated with tumor progression in ovarian cancer, these findings may have therapeutic implications [42]. Although both Compound 3K and shikonin potentiated the effects of cisplatin, shikonin appeared to exert greater inhibitory effects on cell growth, migration, and metabolic activity; however, direct statistical comparisons between the two PKM2 inhibitors were not performed. These differences may reflect the broader range of molecular targets and biological activities of shikonin beyond PKM2 inhibition. Although the combination of shikonin and cisplatin significantly reduced the migratory capacity of SKOV-3 cells, the contribution of enhanced cytotoxicity to this effect cannot be excluded. Therefore, further studies using experimental conditions that minimize the influence of cytotoxicity are warranted to determine whether PKM2 inhibition directly suppresses cell migration.

Finally, the effect of PKM2 inhibition on cellular metabolism was assessed by measuring the extracellular acidification rate (ECAR), a surrogate marker of glycolytic activity [43]. Combination treatment with PKM2 inhibitors and cisplatin significantly reduced glycolytic capacity and glycolytic reserve, consistent with suppression of glycolytic metabolism following PKM2 inhibition. Interestingly, shikonin alone showed a tendency to increase certain glycolytic parameters under the experimental conditions used in this study, whereas compound 3K did not produce a comparable effect. The biological significance of this observation remains uncertain and warrants further investigation. One possible explanation is that shikonin induces a compensatory metabolic response to cellular stress or exerts additional biological effects independent of PKM2 inhibition. Given the complexity of metabolic regulation in cancer cells, further studies are required to elucidate the mechanisms underlying these differential effects. Our findings suggest that PKM2 inhibition alters glycolytic function, as reflected by reduced glycolytic capacity and glycolytic reserve. Although the precise relationship between these metabolic changes and enhanced cisplatin sensitivity remains to be determined, metabolic reprogramming may contribute to the antitumor effects observed in this study. These results provide a rationale for further investigation of PKM2-targeted metabolic strategies for overcoming cisplatin resistance in ovarian cancer. Previous studies have shown that PKM2 overexpression is associated with enhanced glycolysis, increased anabolic metabolism, and resistance to anticancer agents, whereas PKM2 inhibition has been reported to restore chemosensitivity in several tumor types [44,45]. In agreement with these observations, our findings suggest that PKM2 inhibition alters glycolytic function and enhances the response of SKOV-3 cells to cisplatin.

In summary, the findings of this study highlight the potential of combining PKM2 inhibitors with cisplatin as a promising therapeutic strategy for overcoming cisplatin resistance in ovarian cancer. PKM2 inhibition enhanced the anticancer activity of cisplatin by promoting apoptosis, inducing G2/M phase arrest, suppressing cell migration, modulating autophagic responses, and disrupting cellular metabolism in SKOV-3 cells. Collectively, these results suggest that targeting PKM2 may improve the efficacy of cisplatin-based chemotherapy and warrant further preclinical and clinical studies to evaluate the therapeutic potential of PKM2-targeted strategies in ovarian cancer.

## 4. Materials and Methods

### 4.1. Reagents

Cisplatin and shikonin were purchased from Sigma-Aldrich (St. Louis, MO, USA). Cisplatin was prepared in Dulbecco’s phosphate-buffered saline (DPBS) at 2 mM concentration and stored at 4 °C. Shikonin was dissolved in sterile dimethyl sulfoxide (DMSO) and stored at −20 °C. Stock solutions of shikonin were prepared at 10 mM concentration. RPMI 1640 medium (Roswell Park Memorial Institute) and certified heat-inactivated fetal bovine serum (FBS) were obtained from Welgene (Daegu, Republic of Korea). Antibiotic–antimycotics were purchased from Gibco Invitrogen Corporation (Carlsbad, CA, USA). 3-(4,5-dimethylthiazol-2-yl)-2,5-diphenyl-tetrazolium bromide (MTT) was obtained from Alfa Aesar (Ward Hill, MA, USA). Acridine orange was purchased from Sigma-Aldrich.

### 4.2. Cell Lines and Culture Condition

Human ovarian cancer epithelial cells (SKOV-3) were purchased from the American Type Culture Collection (ATCC, HTB-77, Manassas, VA, USA) and cultured using RPMI 1640 medium supplemented with 10% FBS and 1% antibiotic–antimycotics in a humidified incubator at 37 °C with 5% CO_2_. Subculturing was performed every 2–3 d. Additional ovarian cancer cell lines, including TOV-21G (CRL-3577), CAOV-3 (HTB-75), and OVCAR-3 (HTB-161), were obtained from the ATCC (Manassas, VA, USA).

### 4.3. Cytotoxicity Assay

Cytotoxicity was assessed using the MTT assay. Cells (3 × 10^3^ per well) were seeded into a 96-well plate. After incubation for 24 h, the cells were treated with the drugs and incubated for another 24 h. The MTT solution was prepared (1 mg/mL) in DPBS and stored at −20 °C until use. At the endpoint, 100 μL 1× MTT (1 mg/mL) solution was added to each well. The plate was covered and incubated for 3 h in a humidified incubator at 37 °C. After removing the old media, DMSO was added to dissolve the formazan crystals, and the plate was incubated for another 20 min at 37 °C incubator. The absorbance at 540 nm was measured using a VERSA Max Microplate Reader (Molecular Devices Corp., San Jose, CA, USA). Cell viability was normalized to the vehicle-treated control (100%), and IC_50_ values were calculated by fitting the normalized dose–response data from at least three independent experiments using a four-parameter logistic (variable-slope) nonlinear regression model in GraphPad Prism (GraphPad Software, San Diego, CA, USA). The IC_50_ values were determined from the best-fit curve rather than by visual interpolation of individual data points.

### 4.4. Annexin V–FITC/Propidium Iodide Staining Assay

The Annexin V–FITC binding assay was performed using the Annexin V–FITC Staining Kit I (BD Biosciences, San Diego, CA, USA). SKOV-3 cells (2 × 10^5^) were incubated in a 60 mm cell culture dish for 24 h and treated with shikonin (1 and 1.5 μM), or 0.1% DMSO (vehicle control) for 24 h. Then, the cells were harvested, and 5 μL Annexin V–FITC and propidium iodide (PI) were added to 100 μL binding buffer per sample, according to the manufacturer’s instructions. The samples were then incubated in dark for 30 min at 24 °C, diluted with 500 μL DPBS, and analyzed using the Novocyte Flow Cytometer (ACEA Biosciences, San Diego, CA, USA).

### 4.5. Cell Cycle Analysis

Cells (6 × 10^5^) were seeded in a 100 mm cell culture dish at 70% confluency and incubated for 24 h. The cells were then treated with shikonin (1 and 1.5 μM), or 0.1% DMSO (vehicle control). After 24 h, the cells were harvested and fixed overnight with 70% ethanol at −20 °C. For staining, 5 μL 1 mg/mL PI and 10 μL 10 mg/mL RNase A were added to 500 μL DPBS per sample. The samples were then incubated in dark for 30 min at room temperature. Cell cycle analysis was performed using the Novocyte Flow Cytometer.

### 4.6. Wound Healing Assay

Cells (2 × 10^4^ cells) were seeded in a 96-well culture plate to achieve 100% confluency during treatment. After 24 h, a single scratch was made in the middle of each well using a 96-well pin round marker. The scratched area was washed with DPBS, and the cells were treated with various shikonin concentrations. The same spots on the plates were photographed every 12 h using a Cytation Cell Imaging Reader (BioTek Instruments, Winooski, VT, USA). After each use, the scratched device was cleaned with 1% Virkon and 0.5% Alconox. For data analysis, the initial scratched area was considered 0% and the completely closed area was set to 100%. The extent of cell migration filling the scratch area over time was analyzed using Cytation, and the data were graphed using GraphPad Prism7.

### 4.7. Acridine Orange Staining

Cells (1.5 × 10^4^ cells/well) were seeded in a confocal dish to achieve 70% confluency and incubated for 24 h. The cells were treated with shikonin (1 μM) or 0.1% DMSO (vehicle control). After 24 h, the cells were fixed with 4% paraformaldehyde for 15 min and washed with DPBS. The cells were stained with 1 μg/mL acridine orange for 15 min, followed by washing with cold DPBS to remove any excess dye from the background. The stained cells were examined under a K1-Fluo microscope (Nanoscope Systems, Daejeon, Republic of Korea) at 400× magnification.

### 4.8. Seahorse XF Glycolysis Stress Test

For Seahorse XF analysis, drug concentrations were selected based on preliminary optimization experiments to minimize acute cytotoxicity while allowing reliable detection of metabolic changes during the assay period. Cells (2 × 10^3^ cells) were seeded in a Seahorse 96-well XF cell culture microplate. After 24 h, the cells were treated with shikonin (0.5 and 1 μM), cisplatin (20 μM), or 0.1% DMSO and 1% DPBS (vehicle control) for 24 h. The sensor cartridge of the Seahorse XFe96 Extracellular Flux Assay Kit (Agilent Seahorse, Billerica, MA, USA) was hydrated the day before the assay by placing it in sterile water at 37 °C. On the day of the assay, the sensor cartridge was calibrated using a calibrant solution following the manufacturer’s instructions. For the assay, 10 mM glucose, 1.0 μM oligomycin, and 50 mM 2-deoxyglucose (2-DG) were loaded to the sensor wells. The calibrated sensor cartridge was then placed on a cell culture microplate and inserted into an Agilent Seahorse XF analyzer (Agilent Seahorse, Billerica, MA, USA). A graph of the extracellular acidification rate (ECAR) was obtained to provide insight into the cellular metabolic activity.

### 4.9. Western Blot Analysis

Cells (4.5 × 10^5^) were seeded in a 100 mm cell culture dish. After 24 h, the cells were treated with shikonin, cisplatin, or 0.1% DMSO and 1% DPBS (vehicle control) and incubated at 37 °C for 24 h. The cells were harvested, washed with cold DPBS to remove any remaining media, and resuspended in Pro-Prep lysis buffer to extract the total protein. The protein concentration was quantified using the BCA protein assay, with bovine serum albumin (BSA) as the standard. Protein samples were adjusted to 10 μg/mL for loading, separated by sodium dodecyl sulfate–polyacrylamide gel electrophoresis (SDS–PAGE), and transferred on a polyvinylidene difluoride (PVDF) membrane. After transfer, the membrane was incubated in a blocking solution (BSA or skim milk) for 1 h at room temperature and then incubated overnight at 4 °C with the primary antibody with gentle agitation on a shaker. After incubation with the primary antibody, the membrane was washed with Tris-buffered saline containing 0.1% Tween 20 (TBST) and incubated with a horseradish peroxidase-conjugated secondary antibody for 2 h at room temperature on a shaker. The membrane was washed again with TBST, and the protein bands were visualized by spraying with Immobilon HRP Substrate Forte. Luminescent signals were detected using the ChemiDoc Imaging System. Protein expression levels were quantified using ImageJ software (1.54t), and band intensity was expressed as a bar graph.

### 4.10. Statistical Analysis

Data are expressed as mean ± standard deviation (SD). Statistical analyses were conducted using GraphPad Prism software (version 8.0). Statistical significance was determined by one-way ANOVA followed by Tukey’s post hoc test. Survival curves were compared using the log-rank (Mantel–Cox) test. A *p* value of less than 0.05 was considered statistically significant.

## Figures and Tables

**Figure 1:**
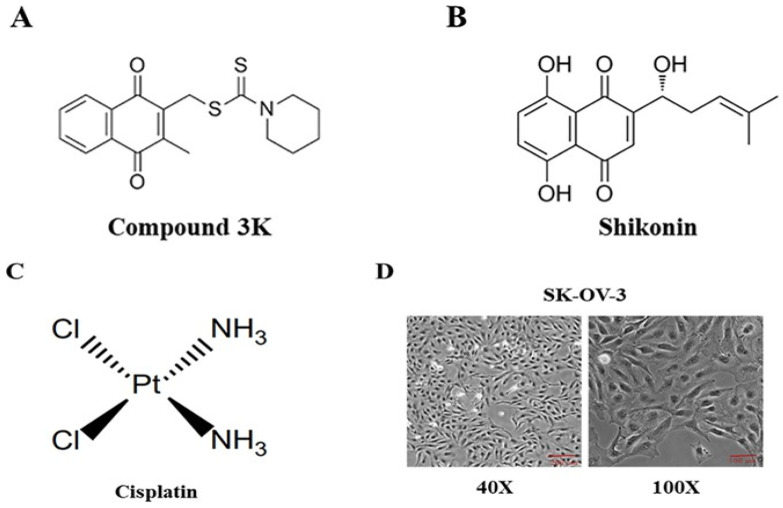
Chemical structures of pyruvate kinase M2 (PKM2) inhibitors and cisplatin and SKOV-3 cell morphology. (**A**,**B**) The chemical structure of PKM2 inhibitors, compound 3K and shikonin. (**C**) The chemical structure of anticancer drug, cisplatin. (**D**) Representative microscopic images are shown with scale bars indicating the corresponding dimensions. Magnification information has been verified and corrected where necessary. Scale bar, 200 μm (40×), 100 μm (100×).

**Figure 2:**
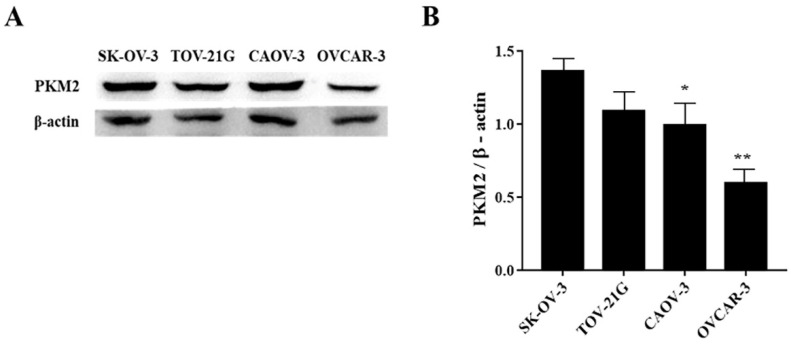
Pyruvate kinase M2 (PKM2) expression levels in various ovarian cancer cell lines. (**A**) PKM2 protein expression levels in ovarian adenocarcinoma cell lines were analyzed by Western blot. Wild-type ovarian cancer cell lines, including SKOV-3, TOV-21G, CAOV-3, and OVCAR-3 show different PKM2 expression levels. (**B**) The band intensities from (**A**) are quantified and presented as a bar graph, expressed as the ratio of PKM2 normalized to β-actin. All data are expressed as mean ± standard deviation (SD) of three independent experiments. Statistical significance was determined by one-way ANOVA followed by Tukey’s post hoc test * *p* < 0.05, ** *p* < 0.01.

**Figure 3:**
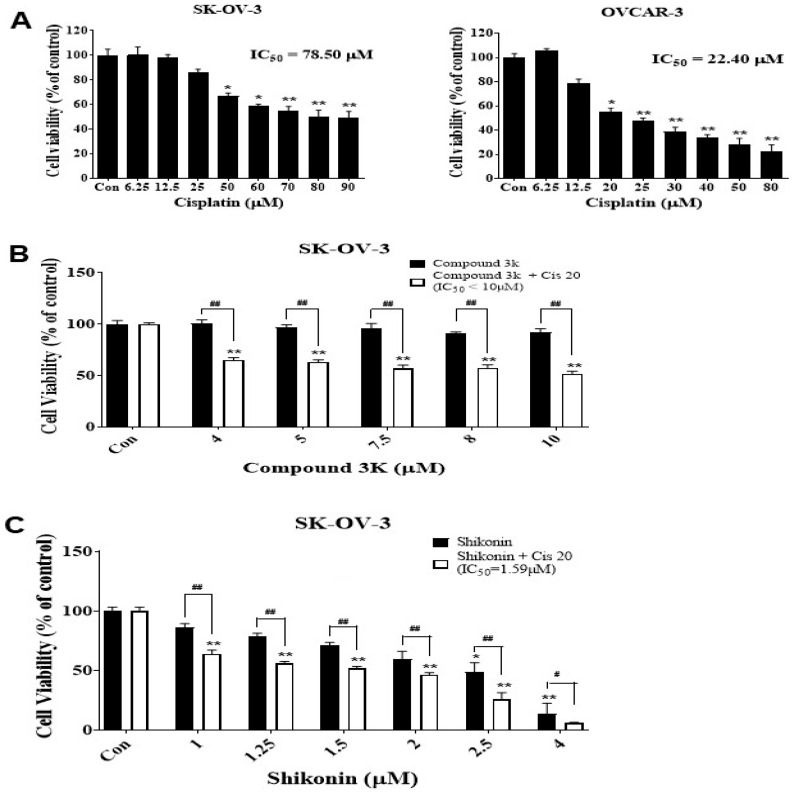
Effects of PKM2 inhibitors on cell viability and cisplatin sensitivity in ovarian cancer cells. (**A**) Cell viability of SKOV-3 and OVCAR-3 cells following treatment with increasing concentrations of cisplatin for 24 h. Cell viability was determined using the MTT assay, and IC_50_ values were calculated by fitting the normalized dose–response data from at least three independent experiments using a four-parameter logistic (variable-slope) nonlinear regression model in GraphPad Prism (GraphPad Software, San Diego, CA, USA). (**B**) Effects of Compound 3K, alone or in combination with cisplatin (20 μM), on the viability of SKOV-3 cells. Cells were treated with the indicated concentrations of Compound 3K for 24 h, and cell viability was measured by the MTT assay. (**C**) Effects of shikonin, alone or in combination with cisplatin (20 μM), on the viability of SKOV-3 cells. Cells were treated with the indicated concentrations of shikonin for 24 h, and cell viability was assessed by the MTT assay. Data are presented as the mean ± SD from three independent experiments. Statistical significance was analyzed using one-way ANOVA followed by Tukey’s multiple comparisons test. ^#^ *p* < 0.05 and ^##^ *p* < 0.01 versus the corresponding untreated control group. For the combination treatment groups, statistical comparisons were performed between cisplatin alone and the corresponding cisplatin plus PKM2 inhibitor treatment at each inhibitor concentration. * *p* < 0.05 and ** *p* < 0.01 versus the corresponding cisplatin-treated group.

**Figure 4:**
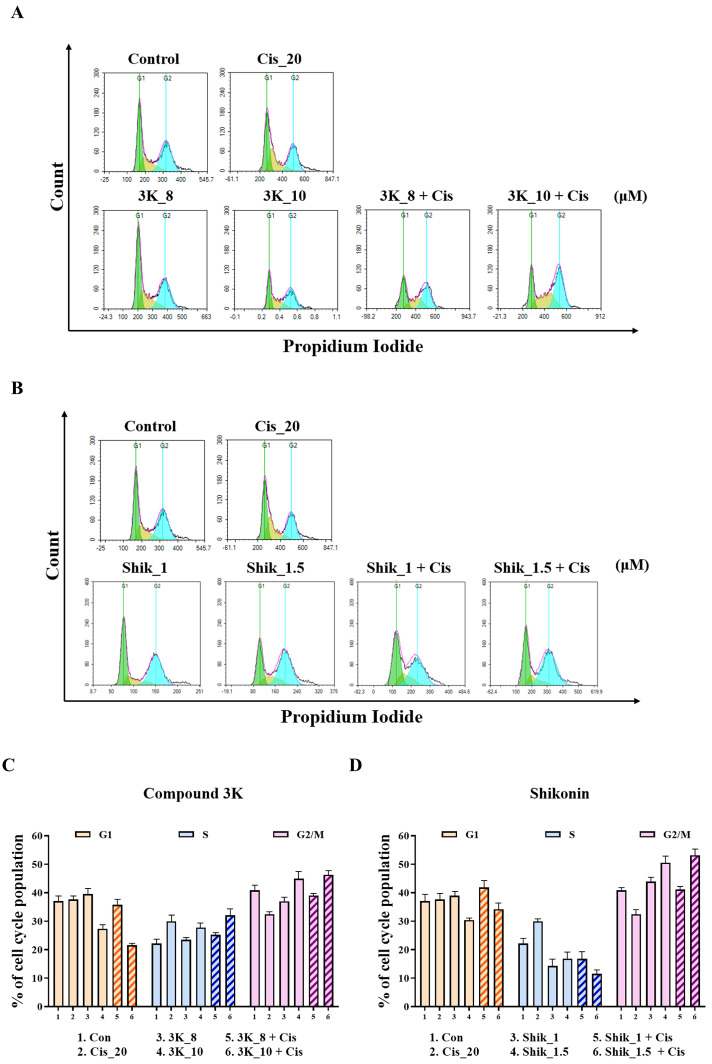
Effects of PKM2 inhibitors on cell−cycle distribution in cisplatin-resistant SKOV−3 cells. (**A**,**B**) Representative flow cytometric histograms of propidium iodide (PI)−stained SKOV−3 cells following treatment with Compound 3K (**A**) or shikonin (**B**), either alone or in combination with cisplatin, for 24 h. Cell−cycle distribution was analyzed by flow cytometry after PI staining. Green: G1 phase, Blue: G2 phase, and Yellow: S phase. (**C**,**D**) Quantitative analysis of the percentages of cells in the G0/G1, S, and G2/M phases following treatment with Compound 3K (**C**) or shikonin (**D**), either alone or in combination with cisplatin. Data are presented as the mean ± SD from three independent experiments.

**Figure 5:**
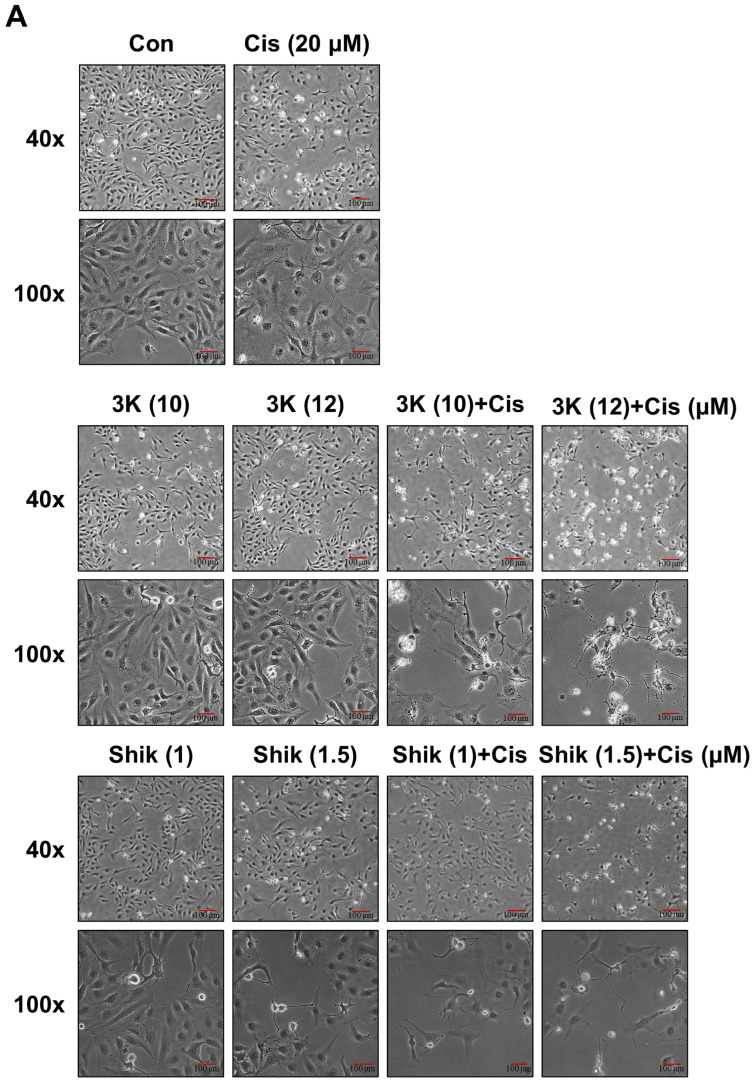

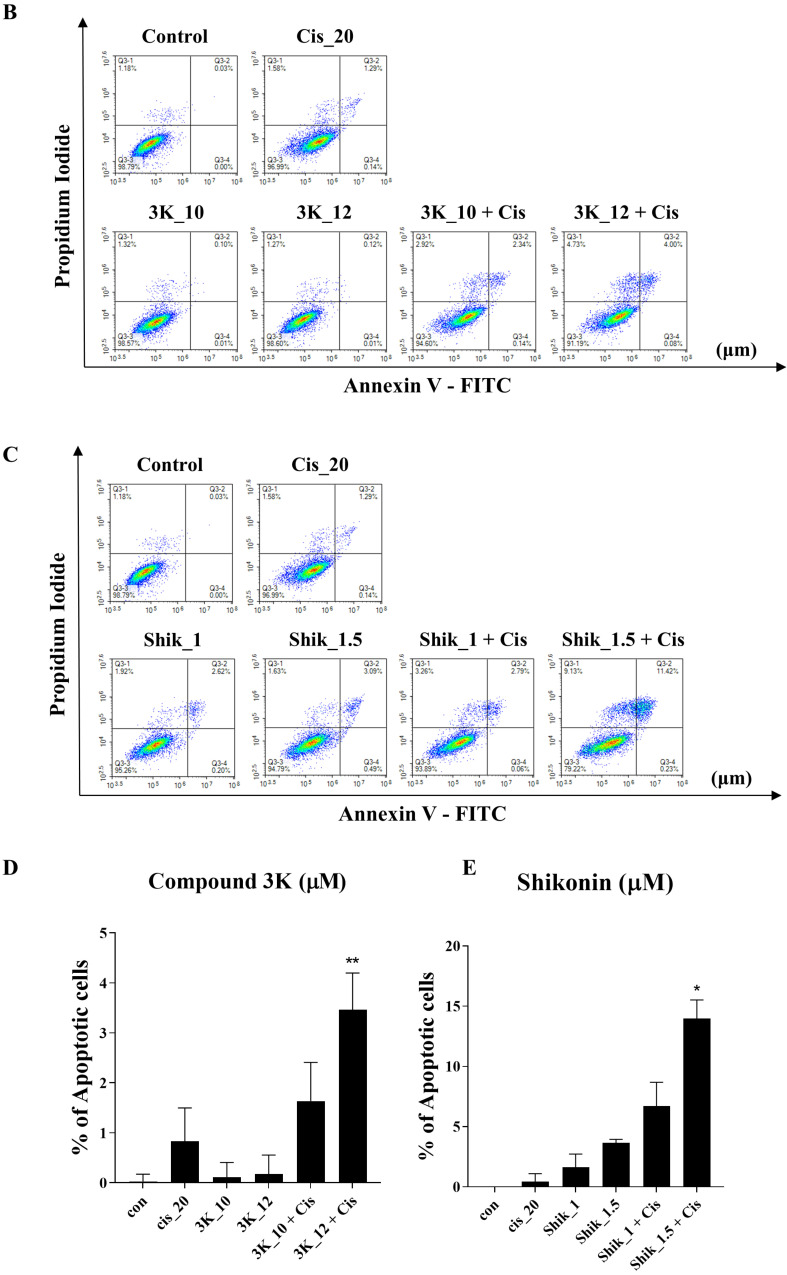
Effects of PKM2 inhibitors on morphological changes and apoptosis in SKOV-3 cells following treatment with PKM2 inhibitors and cisplatin. (**A**) Representative phase-contrast images of SKOV-3 cells treated with cisplatin (20 μM), Compound 3K (10 or 12 μM), or shikonin (1 or 1.5 μM), either alone or in combination, for 24 h. Images were acquired at ×40 and ×100 magnification. Treatment with Compound 3K or shikonin alone produced modest morphological changes, whereas combination treatment with cisplatin resulted in a marked reduction in cell density and an increase in rounded and detached cells, consistent with enhanced cytotoxicity. Scale bars are shown in each image. Scale bars = 200 μm (40×) and 100 μm (100×). (**B**,**C**) Representative Annexin V-FITC/propidium iodide (PI) flow cytometric dot plots of SKOV-3 cells treated with Compound 3K (**B**) or shikonin (**C**), either alone or in combination with cisplatin, for 24 h. Apoptotic cells were quantified as the sum of early apoptotic (Annexin V-positive/PI-negative) and late apoptotic (Annexin V-positive/PI-positive) populations. (**D**,**E**) Quantitative analysis of total apoptotic cells following treatment with Compound 3K (**D**) or shikonin (**E**), either alone or in combination with cisplatin. Data are expressed as mean ± SD of three independent experiments. Statistical significance was determined by one-way ANOVA followed by Tukey’s post hoc test * *p* < 0.05, ** *p* < 0.01.

**Figure 6:**
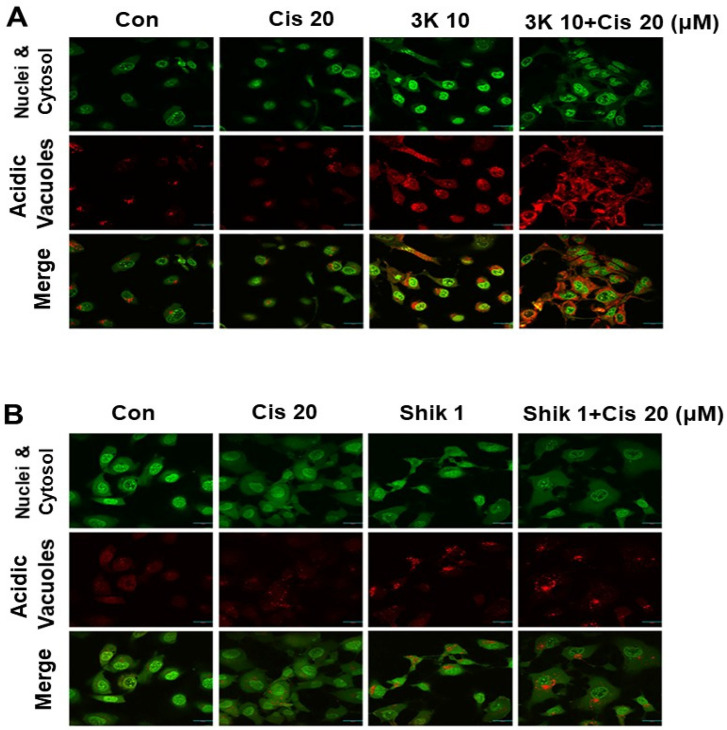
Acridine orange (AO) staining of SKOV-3 cells following treatment with PKM2 inhibitors and cisplatin. (**A**,**B**) SKOV-3 cells were treated with Compound 3K (10 μM), shikonin (1 μM), cisplatin (20 μM), or their combinations for 24 h and stained with acridine orange to evaluate the formation of acidic vesicular organelles (AVOs), an indicator of autophagic activity. Green fluorescence represents the nuclei and cytoplasm, whereas red fluorescence indicates acidic vesicular organelles, including autophagosomes and autolysosomes. Representative confocal fluorescence images were acquired at ×400 magnification. Representative confocal microscopy images are shown with preserved aspect ratios. Scale bars = 100 μm.

**Figure 7:**
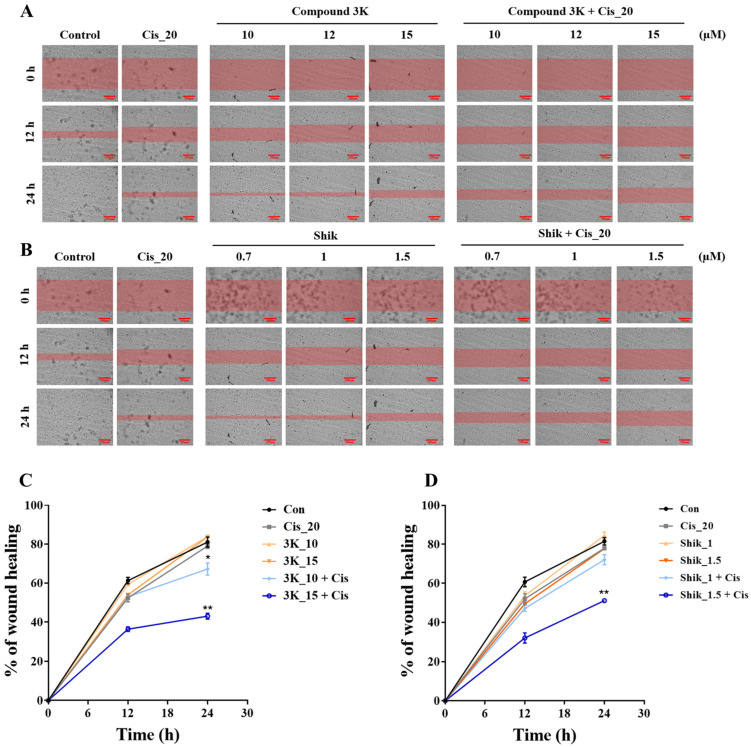
SKOV-3 cell migration assay after treatment with pyruvate kinase M2 (PKM2) inhibitors and cisplatin. Representative wound-healing images are shown with preserved aspect ratios and scale bars = 200 μm. Images were acquired using a Cytation imaging system at 200× magnification. Wound boundaries are indicated for visualization purposes and have been adjusted to minimize obstruction of the underlying image. (**A**) Cells were treated with the compound 3K (10 or 15 μM) and cisplatin (20 μM). The cell migration ability was confirmed at 12 h intervals for 24 h. (**B**) Cells were treated with the shikonin (1 or 1.5 μM) and cisplatin (20 μM). The red areas indicate the automatically detected by the IncuCyte to measure cell migration. (**C**,**D**) Quantitative migration data are presented as mean ± SD from three independent experiments. Statistical significance was determined by one-way ANOVA followed by Tukey’s post hoc test * *p* < 0.05, ** *p* < 0.01.

**Figure 8:**
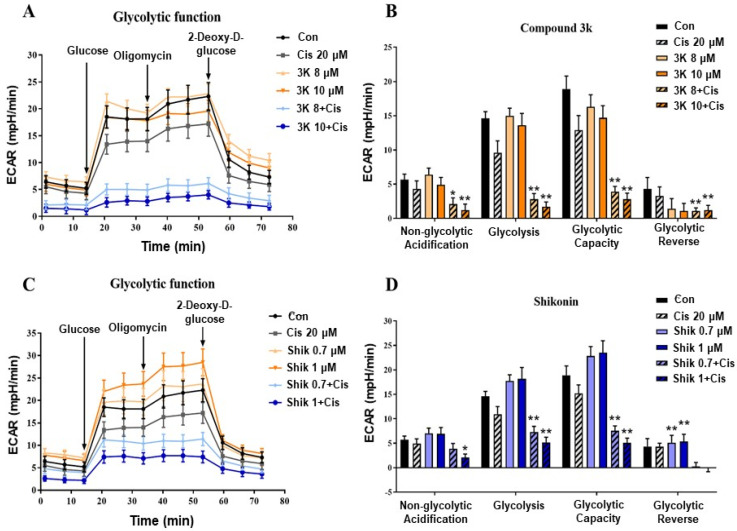
Measurement of the extracellular acidification rate (ECAR) for glycolytic functional stress after pyruvate kinase M2 (PKM2) inhibition. Basal glycolysis was calculated as the increase in ECAR after glucose treatment. Glycolytic capacity was calculated as the maximum ECAR following oligomycin treatment, and glycolytic reserve was determined as the difference between glycolytic capacity and basal glycolysis. (**A**) ECAR measurement shows the glycolysis functional stress after the treatment with compound 3K (8 or 10 μM) and cisplatin (20 μM) for 24 h. Glucose, oligomycin, and 2−DG were injected to the 96−well cell culture plate automatically. (**B**) From the left, the bar graph shows the non-glycolytic acidification, glycolysis ability, glycolytic capacity, and glycolytic reverse. (**C**) ECAR measurement after treatment with shikonin (0.7 or 1 μM) and cisplatin (20 μM) for 24 h. (**D**) ECAR measurement shows the glycolytic functional stress after combination. Data are expressed as mean ± SD of three independent experiments. Statistical significance was determined by one-way ANOVA followed by Tukey’s post hoc test * *p* < 0.05, ** *p* < 0.01.

## Data Availability

The datasets used and/or analyzed during the current study are available from the corresponding author upon reasonable request.

## Abbreviations

The following abbreviations are used in this manuscript

AMPK	AMP-activated protein kinase
DMSO	Dimethyl sulfoxide
DPBS	Dulbecco’s Phosphate-Buffered Saline
ECAR	Extracellular Acidification Rate
FBS	Fetal bovine serum
PKM2	Pyruvate kinase M2
PVDF	polyvinylidene difluoride (PVDF)
PI	Propidium iodide
mTOR	mammalian/mechanistic Target of Rapamycin
